# A Chinese multi-modal neuroimaging data release for increasing diversity of human brain mapping

**DOI:** 10.1038/s41597-022-01413-3

**Published:** 2022-06-09

**Authors:** Peng Gao, Hao-Ming Dong, Si-Man Liu, Xue-Ru Fan, Chao Jiang, Yin-Shan Wang, Daniel Margulies, Hai-Fang Li, Xi-Nian Zuo

**Affiliations:** 1 College of Information and Computer, Taiyuan University of Technology, Taiyuan, 030024 China; 2 State Key Laboratory of Cognitive Neuroscience and Learning, Beijing Normal University, Beijing, 100875 China; 3 National Basic Science Data Center, Beijing, 100109 China; 4 Institute of Psychology, Chinese Academy of Sciences, Beijing, 100101 China; 5 School of Psychology, Capital Normal University, Beijing, 100048 China; 6 Centre National de la Recherche Scientifique, Frontlab, Brain and Spinal Cord Institute, Paris, UMR 7225 France; 7 Developmental Population Neuroscience Research Center, IDG/McGovern Institute for Brain Research, Beijing Normal University, Beijing, 100875 China; 8 Key Laboratory of Brain and Education, School of Education Science, Nanning Normal University, Nanning, 530001 China

**Keywords:** Cognitive neuroscience, Psychology

## Abstract

The big-data use is becoming a standard practice in the neuroimaging field through data-sharing initiatives. It is important for the community to realize that such open science effort must protect personal, especially facial information when raw neuroimaging data are shared. An ideal tool for the face anonymization should not disturb subsequent brain tissue extraction and further morphological measurements. Using the high-resolution head images from magnetic resonance imaging (MRI) of 215 healthy Chinese, we discovered and validated a template effect on the face anonymization. Improved facial anonymization was achieved when the Chinese head templates but not the Western templates were applied to obscure the faces of Chinese brain images. This finding has critical implications for international brain imaging data-sharing. To facilitate the further investigation of potential culture-related impacts on and increase diversity of data-sharing for the human brain mapping, we released the 215 Chinese multi-modal MRI data into a database for imaging Chinese young brains, namely’I See your Brains (ISYB)’, to the public via the Science Data Bank (10.11922/sciencedb.00740).

## Abstract (summary)

**Table:** 

Measurement(s)	brain imaging measurements
Technology Type(s)	magnetic resonance imaging
Factor Type(s)	multimodal neuroimaging metrics
Sample Characteristic - Organism	Homo
Sample Characteristic - Environment	magnetic
Sample Characteristic - Location	North China

## Background & Summary

With the worldwide increase in the acquisition of neuroimaging data using magnetic resonance imaging (MRI), the use of big data is becoming a standard practice in the field through international data-sharing initiatives^1–4^. Data sharing can bring many benefits to neuroscience research and increase sample sizes, which allow for greater precision and improve the statistical power to detect smaller effects, although smaller effects may be associated with either smaller biological effects or potentially confounding factors^5^. This has accelerated many fields into their new stages or even entirely new field, such as the developmental population neuroscience^6,7^ or statistical connectomics^8^. On the one hand, it can facilitate researchers to conduct test-retest studies when more neuroimaging public databases are available and improve the reproducibility of research results^9,10^. Data reuse can also make peer review better^9,11^. On the other hand, it encourages cooperation and communication between institutions and organizations, and thus creates a win-win situation for both researchers giving and receiving data^12^.

As the concept of sharing data continues to grow, more and more public databases are emerging to support researchers such as the Human Connectome Project (HCP)^13^ and its derivatives across human lifespan^14^ (e.g., the Adolescent Brain Cognition Development, ABCD^15^), UK Biobank^16^, the Autism Brain Imaging Data Exchange (ABIDE)^17,18^, the 1000 Functional Connectomes Project^19^. These efforts on big-data sharing has propelled the cognitive neurology field forward (see more data projects from our recent reviews^20,21^). However, most of the existing databases are based on Caucasian populations. This preventing us from identifying ethnic or culture-related differences in neuroscience associations. Moreover, China has the largest population in the world and there is still a need to expand and improve the public database of the Chinese brain mapping (few exceptions, e.g., the Chinese Color Nest Project^20^, the REST-meta-MDD project^22^, the Chinese Human Connectome Project^23^ and the Consortium for Reliability and Reproducibility^9^).

Much like the genetics field^24^, data-sharing faces many challenges in the neuroscience field. One of the challenges is to ensure that personal information, especially facial information, remains protected when raw MRI data are shared. This is especially challenging for high-resolution T1-weighted anatomical images, in which facial information has been fully embedded. The higher the image resolution is, the clearer the facial information becomes. It has been possible to restore subject’s facial information through the combination of existing facial recognition and reconstruction technologies. As a result, personal information can easily be disclosed. Therefore, we must first remove or obscure the facial features in brain images, especially in high-resolution structural MRI images. In response to this, the Health Insurance Portability and Accountability Act (HIPAA) of the United States specifically stipulates that the subject’s private photos or equivalent images must be removed from the medical images to protect their privacy^25^. The European Union has general data protection, which requires research institutions to strengthen the privacy protection of personal data. Therefore, data protection should be considered and implemented during the design phase of scientific studies^24,26^.

In order to ensure participant’s private security, all the aforementioned face masking algorithms are involved in taking a standard brain template as references, so as to blur the facial information in the template space. However, to date, previous studies have not dealt with whether a different facial mask will be generated due to a different ethnic template used during anonymization. This may reflect the situation that the existing public brain imaging databases rarely contain non-western samples. In this data descriptor, we release the’I See Your Brains’ database for imaging Chinese young brains and demonstrate the template effects on face masking using head templates of different races. Specifically, ethnically diverse brain templates are employed to anonymize Chinese multimodal MRI data independently. The discrepancy between Chinese brain images anonymized using different templates of race is quantitatively examined. We hypothesized that the use of different (Chinese versus Western) templates will cause significant differences in the face masking performance. Use of the corresponding ethnic template will achieve a better performance in face anonymization.

## Methods

### Participant recruitment

The database, Imaging Chinese Young Brains (namely, “I See Your Brains”, ISYB), is generated by the Institute of Psychology, Chinese Academy of Sciences (IPCAS), which is one contributing site of the Chinese imaging genetics cohort (CHIMGEN)^27^ to enhance cross-ethnic and cross-geographic brain research. It is the largest prospective neuroimaging genetic cohort for Chinese Han adults with natural and socioeconomic measurements obtained from remote sensing. In accordance with CHIMGEN, all participants in ISYB were recruited by advertisements posted in colleges and communities. Participants were excluded if they met any of the following criteria: regular smoker, pregnancy, abnormal color discrimination, a history of alcohol or drug abuse, currently any medication, MRI contraindications, neuropsychiatric or severe somatic disorder and sedative-hypnotic medication within a month or any medication for major neuropsychiatric disorders. The ISYB study was approved by the IPCAS ethical committee and written informed consent was obtained from each participant.

### Image acquisition

ISYB contains multi-modal neuroimaging data from 241 right-handed Chinese healthy volunteers. Each participant received MRI scans of the brain images at a 3.0 Tesla MRI scanner (GE MR750) at the IPCAS MRI Research Center including: (1) the high-resolution T1-weighted structural MRI (sMRI, matrix = 256 × 256, number of slices = 188, field of view = 256 × 256 mm, repetition time = 8.16 ms, echo time = 3.18 ms, flip angle = 12°, inversion time = 450 ms), (2) resting-state functional MRI (rfMRI, matrix = 64 × 64, number of slices = 36, field of view = 220 × 220 mm, repetition time = 2000ms, echo time = 30 ms, flip angle = 90°), (3) diffusion tensor MRI (dMRI, matrix = 128 × 128, number of slices = 50, field of view = 256 × 256 mm, repetition time = 6000 ms, echo time = 65 ms, flip angle = 90°, 64 diffusion-sensitisation directions at b = 1000 with b = 0), and (4) arterial spin labeling MRI (aMRI, matrix = 128 × 128, number of slices = 50, field of view = 240 × 240 mm, repetition time = 5046 ms, echo time = 11.09 ms, flip angle = 111°, inversion time = 2025 ms).

### Quality assurance

All participants were included in the first-step quality assessments of the neuroimaging data. Their anatomical T1 sMRI images were visually inspected to ensure no substantial head motion and structural abnormalities. Two participants were excluded for excessive head motion. In the second-step quality assessments, the T1 sMRI and rfMRI image quality of ISYB datasets were examined using the **MRIQC** toolkit (https://github.com/poldracklab/mriqc), which incorporated a series of quantitative metrics as in the following list. More details of these metrics can be found in the previous work^28,29^.

- Spatial Metrics (sMRI, rfMRI):

1. Signal-to-Noise Ratio (SNR)^30^: The mean value of gray matter divided by the standard deviation (SD) of the air.
2. Foreground to Background Energy Ratio (FBER): Mean energy of image values (i.e., mean of squares) within the head relative to outside the head.
3. Contrast-to-Noise Ratio (CNR) (only sMRI)^30^: Calculated as the mean of the gray matter values minus the mean of the white matter values, divided by the standard deviation of the air values.
4. Entropy Focus Criteria (EFC)^31^: Shannon’s entropy is used to summarize the principal directions distribution.
5. Smoothness of Voxels^32^: The full-width half maximum (FWHM) of the spatial distribution of image intensities.

- Temporal Metrics (rfMRI):
  1. Mean Framewise Displacement (FD)^33^: A measure of subject head motion, which compares the motion between the current and previous volumes. This is calculated by summing the absolute value of displacement changes in the x, y and z directions and rotational changes about those three axes. The rotational changes are given distance values based on the changes across the surface of a 50 mm radius sphere.
  2. Standardized DVARS^33^: The spatial standard deviation of the temporal derivative of the data (D referring to temporal derivative of time series, VARS referring to root-mean-square variance over voxels), normalized by the temporal standard deviation and temporal auto-correlation.
  3. Global Correlation (GCOR)^34^: The average of the entire brain correlation matrix, which is computed as the brain-wide average time series correlation over all possible combinations of voxels.

The mean and SD were calculated for each of the eight (five spatial metrics and three temporal metrics) to remove extreme values or outliers which is 3-SD above or below the mean. Taking the effects of head movement in rfMRI into consideration, the data with average FD greater than 0.20 mm^33^ were further excluded. We note that there were 26 participants excluded by the quality assessment procedure, remaining 215 participants included for the subsequent analyses (156 females; 18–30 years old, mean = 22.55 yrs, SD = 2.68 yrs).

**Fig. 1:**
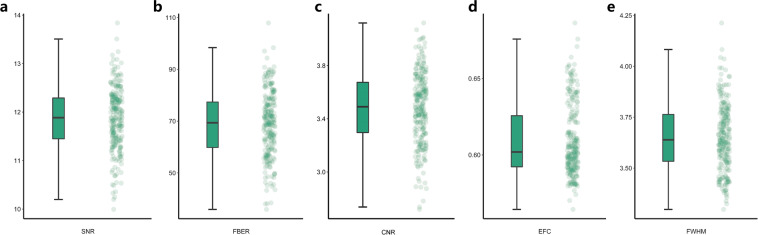
Quality assessments on the ISYB sMRI data using both bar (the left column) and scatter (the right column) plots of the following quality metrics: (**a**) Signal-to-Noise Ratio (SNR) (**b**) Foreground to Background Energy Ratio (FBER) (**c**) Contrast-to-Noise Ratio (CNR) (**d**) Entropy Focus Criteria (EFC) (**e**) Full-Width Half Maximum (FWHM).

**Fig. 2:**
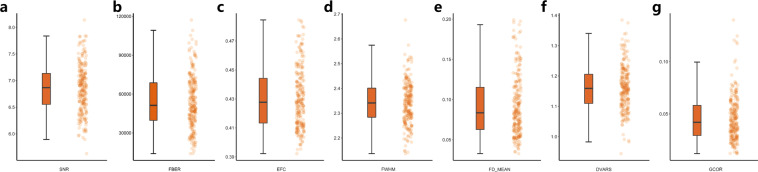
Quality assessments on the ISYB rfMRI data using both bar (the left column) and scatter (the right column) plots of the following quality metrics: (**a**) Signal-to-Noise Ratio (SNR) (**b**) Foreground to Background Energy Ratio (FBER) (**c**) Entropy Focus Criteria (EFC) (**d**) Full-Width Half Maximum (FWHM), (**e**) Mean Framewise Displacement (FD_MEAN), (**f**) Derivative of time series and root-mean-square VARiance over voxelS (DVARS), (**g**) Global Correlation (GCOR).

The assessments on the results of the quality assurance of structural MRI images and functional MRI images are summarized in Figs. 1 and 2, respectively. Details of individual quality reports are presented in the supplementary figures (SFig. 1 for sMRI and SFig. 2 for rfMRI).

### Standard brain templates

Taking into consideration on the global scope of the open science community and data-sharing initiatives, it is critical to realize that the face masking procedure initially uses Western head template as reference. Extensive research has shown that the race factor played a major role when comparing the group differences in brain morphology metrics. For example, previous studies have indicated significant morphological differences between Chinese and Western brains in size^35^, shape^36^, and anatomical volume^37^. Caucasian brains are significantly longer but present a smaller width and a decreased height compared with Chinese brains^23,38^. Thus, taking templates of different races as reference during registration exerts a significant impact on the results of the registration, which may lead to systematic differences in subsequent morphological measurements^39^. Selecting ethnicity-matched templates to analyze structural MRI data may be preferable, which could improve the accuracy of the registration and obtain robust morphological results^40^. This is attributed to the differences in the brain structure between China and the West population, which cause some systematic bias in the registration between individual images and template images and reduce the accuracy in morphological measurements. Such bias has been reproduced in diverse samples across imaging protocols and lifespan studies^41–44^.

Ethnicity-specific brain templates are discrepant regarding the diversity in the brain structures between the East and the West. To test the template effects on face masking, we chose two Chinese brain templates (CN200 and Chinese2020): (1) CN200 is a head template assembled by MRI T1 images using the 3 T Siemens Prisma scanner from 200 healthy Chinese Han participants from the Chinese Human Connectome Project^23^; (2) Chinese2020 is a head template generated based on the MRI T1 images under multiple 1.5 T magnets collected from more than 2,000 healthy participants across multiple centers and regions^45^. The MNI152 head template was used as the Western template, which is the default head template as in the face masking toolkit.

### Face anonymization

In order to effectively blur the part of the face in images and protect privacy as much as possible, researchers have developed several toolkits to remove facial information. For example, Quickshear segments brain images into facial features and brain, and configures the voxels of the facial features to 0, thus resulting in identifiable facial features cut effectively^25^. FreeSurfer determines the location of facial information and creates a brain template after manually separating brain tissue from non-brain tissue. In the subsequent images, the voxel position of non-brain tissue is obtained through the established linear transformation relationship and the voxel value of the facial information position is eliminated^46^. Although these two methods effectively remove facial privacy information, they also lose some areas of the original image at the same time (e.g., intracranial volume and cerebrospinal fluid volume), which may cause the problem of lacking important anatomical features when calculating certain structural metrics. To address this privacy concern, Milchenko and colleagues have developed a deface toolkit, namely face masking, which extracts the anatomical surface of the face in the volume space and then obscures it^47^. The recognition rate of the image is reduced by changing the image resolution, leading to ideal anonymization of the data without disturbing reliability and validity of subsequent tissue extraction and measurements of brain morphology^48–51^. This procedure has become a built-in function of the Extensible Neuroimaging Archive Toolkit^52^ for facial anonymization and been incorporated into the Human Connectome Project^53^. For these reasons mentioned above, when data in the ISYB database were anonymized for facial information, we adopted the CN200 Chinese template. The purpose of which is to remove more privacy and obtain a better anonymization effect for facial information in Chinese brain data. The results and discussion of data anonymization based on different ethnic brain templates are included in Usage Notes session. The ISYB data are processed by the face masking toolkit.

## Data Records

As supports of the open sciences, we have released the multimodal neuroimaging data of the 215 participants in the ISYB database for both research and education in human brain mapping as well as culture association studies. Specifically, as demonstrated in the previous sections, we first applied the face masking pipeline based on CN200 brain template to anonymize the MRI data by blurring facial information while all the participants agreed to share their anonymized data to the public. We then organized the data according to the Brain Imaging Data Structure (BIDS: https://bids.neuroimaging.io)^54^ and employed the **MRIQC** toolkit for monitoring the quality of all structural and functional images. This toolkit generated comprehensive reports on the data quality, which were presented as in the Supplementary Fig. 1 and Supplementary Fig. 2 at group level. All the BIDS-formatted data and related files on the QC reports have been publicly shared through the Science Data Bank (SciDB) at the National Basic Science Data Center (10.11922/sciencedb.00740)^55^.

The *ISYB_DATA* folder contains 215 folders labeled with participant ID numbers (e.g., sub-0002). Each participant’s folder contains subfolders labeled *func* containing the rfMRI images (.nii.gz), *anat* containing defaced T1-weighted sMRI images (.nii.gz), *perf* containing aMRI images (.nii.gz), *dwi* containing dMRI images (.nii.gz,.bval,.bvec). The *ISYB_QC* folder contains QC reports generated by **MRIQC** toolkit for each participant. All QC results were integrated into webpages, and linked in an overview page on group analysis. Of note, the current ISYB release contained the demographic information (age and sex) but did not include any behavioral and clinical measurements.

## Technical Validation

As in the Consortium for Reliability and Reproducibility (CoRR)^9^, we demonstrate the utility of the released ISYB multi-modal MRI data. All the anonymized and quality-controlled data were preprocessed using the Connectome Computation System (CCS^56^, https://github.com/zuoxinian/CCS) with the most recent updates^57^. This pipeline integrates multiple analytical software packages to achieve imaging processing of multi-modal MRI data. Considering the advantages of surface-based functional brain mapping^58^, we reconstructed cortical surface models to generate NIFTI file for structural and functional metrics on the cortical surface. The structural image went through the following preprocessing steps: (1) spatially adaptive non-local means denoising, (2) rough inhomogeneity correction, (3) spatial normalization into the MNI standard brain space, (4) inhomogeneity correction, (5) intensity normalization, (6) brain extraction by non-local intracranial cavity extraction (NICE), and (7) gray and white matter segmentation, surface reconstruction. The rfMRI image preprocessing included (1) dropping off the first 5 EPI volumes, (2) removing and interpolating temporal spikes, (3) correcting acquisition timing among image slices and head motion among image volumes, (4) normalizing the 4D global mean intensity to 10,000, (5) regressing out head motion artifacts and other spurious noise by using ICA-AROMA^59,60^, and (6) removing linear and quadratic trends from the rfMRI signals to mitigate the scanner-related influences^56^.

The preprocessed NIFTI images were converted into the GIFTI format using the Ciftify toolbox^61^. The sMRI images were further preprocessed using the custom pipeline to generate highly refined cortical surface meshes which were spatially normalized to the MNI space and re-sampled to have 32k vertices, i.e., the fsaverage_LR32 cortical surface. Individual dMRI and aMRI images were preprocessed in individual volume space and then transformed onto the fsaverage_LR32 space. Individual surface-mapped rfMRI signals were then brought into the register across participants using a multi-modal surface matching algorithm to the and vectorized into the CIFTI format. This maps each surface vertex to an index in a vector by the Ciftify toolbox. A group-level surface mask was finally established by including every vertex where all the 215 participants obtained the full rfMRI signals. Based on the surface-based data, we derived two sets of brain imaging metrics using CCS for both structural and functional characteristics, respectively. Specifically, following derivatives were calculated:

- Cortical Thickness (CT)^62^: The average distance between the gray/white boundary and the pial surface within each ROI.
- Surface Area (SA)^62^: The sum of the areas of each tessellation falling within each ROI.
- Gray Matter Volume (GMV): The measure of the density of brain cell body in a particular region.
- Local Gyrification Index (LGI)^63^: The ratio of a regional surface area for the pial surface to a smooth surface contour estimated to wraps around the pial surface.
- Fractional Anisotropy (FA)^64^: An index for the amount of diffusion asymmetry within a voxel, defined in terms of its eigenvalues.
- Mean Diffusivity (MD)^64^: The mean amount of diffusion in each of the principal directions calculated in the tensor.
- Regional Homogeneity (ReHo)^58^: The synchronicity of a voxel’s time series and that of its nearest neighbors based on Kendall’s coefficient of concordance to measure the local brain functional homogeneity.
- Voxel-Mirrored Homotopic Connectivity (VMHC)^65^: The functional connectivity between a pair of geometrically symmetric, inter-hemispheric voxels.
- Amplitude of Low-Frequency Fluctuations (ALFF)^66^: The total power in the low frequency range (0.01–0.1 Hz) of an fMRI image, normalized by the total power across all frequencies measured in that same image.
- Cerebral Blood Flow (CBF)^67^: The rate of delivery of arterial blood to the capillary bed in brain tissue and is quantified in milliliters of blood per 100 g of brain tissue per minute.

**Fig. 3:**
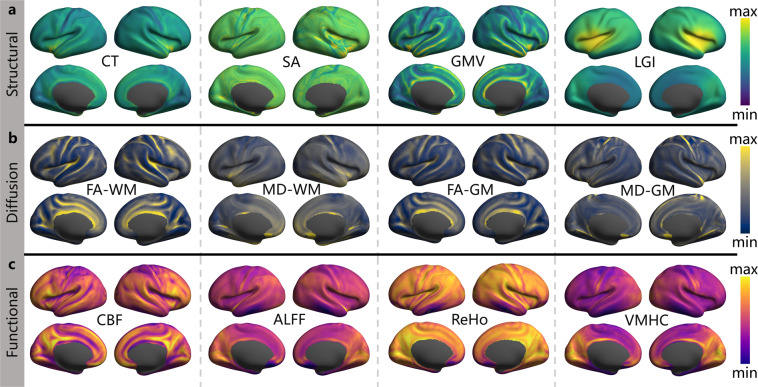
The group mean maps of the ISYB multi-modal neuroimaging data on the fsLR_32k cortical surfaces. Given each vertex on the surface, the mean values were calculated across all the 215 participants for the 12 metrics based on the following three MRI modalities: (**a**) sMRI (CT, SA, GMV, LGI), (**b**) dMRI (FA-WM, MD-WM, FA-GM, MD-GM), (**c**) rfMRI and aMRI metrics (CBF, ALFF, ReHo and VMHC).

**Fig. 4:**
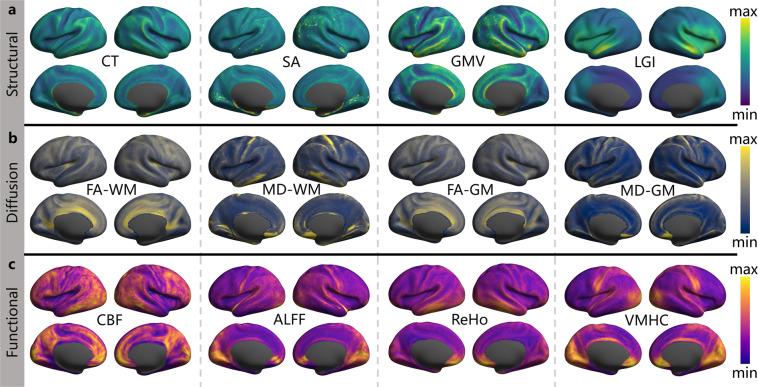
The group variability maps of the ISYB multi-modal neuroimaging data on the fsLR_32k cortical surfaces. Given each vertex on the surface, the standard deviation values were calculated across all the 215 participants for the 12 metrics based on the following three MRI modalities: (**a**) sMRI (CT, SA, GMV, LGI), (**b**) dMRI (FA-WM, MD-WM, FA-GM, MD-GM), (**c**) rfMRI and aMRI metrics (CBF, ALFF, ReHo and VMHC).

We note that each metric was calculated within the group-level surface mask. The CT metric was generated and extracted from the structural outcomes of **ciftify** in the fsaverage_LR32k space. The LGI metric was produced by the FreeSurfer scripts (https://surfer.nmr.mgh.harvard.edu/fswiki/LGI). The SA, ReHo, ALFF and VMHC metrics were calculated by the CCS scripts from the sMRI and rfMRI outcomes of **ciftify**. CCS also provided a computational workflow for dMRI data to derive both FA and MD for gray matter and white matter voxels in individual volume spaces. The results were named as FA-WM, MD-WM, FA-GM and MD-GM, respectively. The **bbregister** command in FreeSurfer was then applied to the native individual maps of CBF, SA, FA and MD to register these individual volume maps onto the fsaverage surface template. The **wb_command -metric-resample** command in the HCP workbench was used to map the native individual CBF, SA, FA and MD metrics to fs_LR and resample them to the 32k space. The vertex-wise group-level maps across all qualified participants are shown on the fsLR_32k surface as in Fig. 3 (mean) and Fig. 4 (SD).

## Usage Notes

We encourage other labs to use this dataset in publication under the requirement of citing this article and contact us for additional data sharing and cooperation. The user of the ISYB database should acknowledge the contributions of the original authors and research lab, and properly cite this article. We note that the impact of the brain template on the facial anonymization procedure is not negligible. In this section, to evaluate the anonymization of different racial brain template, we used high-resolution sMRI images from the database. The ISYB database contains the 215 Chinese multi-modal MRI images^55^ and is part of the CHIMGEN consortium (see more details in the consortium paper^27^). We split the ISYB dataset into two subsets for replicability validation (Discovery sample: ISYB-1, n = 89; Validation sample: ISYB-2, n = 126).

### Implementation of analytic framework

**Fig. 5:**
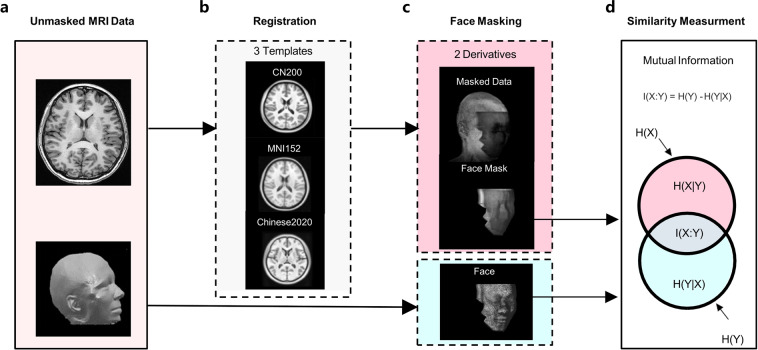
The analytic framework of the evaluation of template effects on face anonymization. (**a**) The face masking toolkit takes raw individual unmasked MRI data with the registration from the unmasked head image to the head template. (**b**) The head templates include the default MNI152 and other two Chinese (CN200 and Chinese2020) templates. (**c**) During the face masking, a face mask is generated and used for restoring the face removed by the face masking pipeline. (**d**) The normalized mutual information between the removed face and the original face is calculated.

As illustrated in Fig. 5, we implemented an analytical framework for evaluation of the template effects on face masking. We noted that the Western brain template (i.e., MNI152 template) was employed as the default brain template in the face masking toolkit. We thus substituted the MNI152 template with the CN200 and Chinese2020 templates to establish the corresponding relationship (i.e., registration) between the two Chinese templates and the individual data space. The corresponding relationship and different ethnic templates were paired and integrated into the face masking process. The three different pipelines were then used to implement the anonymous processing of the MRI data with the different templates. As for the evaluation of the face anonymization performance, we first subtracted the anonymized image from the original image to get the facial information, which was removed from the original face. We then identified the facial areas blurred by the face masking toolkit and created a mask for each blurred surface based on the individual MRI image.

Normalized mutual information (NMI) was used to quantify the efficiency of performing the face-masking process with different templates. NMI is widely used to evaluate the similarity between two sets of information. It serves as an important indicator in information theory and can be used to evaluate the spatial similarity or information shared between images. Its value ranges from 0 to 1. A higher NMI value indicates more spatial information shared between a pair of brain images. In the present study, a higher NMI value reveals more facial information picked up by the face-masking pipeline, and thus more efficient in removing private facial information from the original MRI head image. It means that the privacy information of the participant is blurred more efficiently. Therefore, we hypothesize that the face-masking pipelines with a race-appropriate head template would be more effective and recommendable for the anonymization of the facial information.

**Fig. 6:**
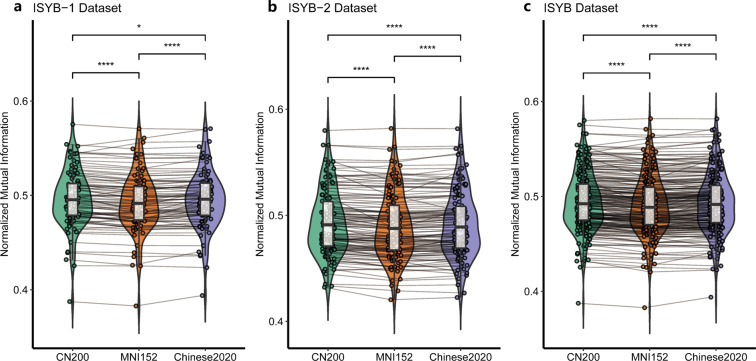
The replicable template effects on face anonymization. Normalized mutual information between the face image from original data and the face image removed by the anonymization are visualized as violin plots with paired lines under the three different template (CN200, MNI152, Chinese2020) conditions. This template effect is replicated across three datasets: (**a**) ISYB-1, (**b**) ISYB-2, (**c**) ISYB. *p<0.05, ****p<0.0001.

When the original brain images from Chinese (ISYB-1 and ISYB-2) passed through the face masking procedure with three different head templates (CN200, Chinese2020 and MNI152), six sets of facial masked images under the 2 × 3 different conditions were generated. The NMI values between the facial information removed under different conditions and the corresponding original data were calculated for each participant. A two-way analysis of variance with repeated measures (database versus template) was performed on the NMI values with the head template as the within-participant factor for testing the interaction effect between template and database on the face masking pipeline. The statistical model revealed a highly significant template effect (*DoF* = 4, *F* = 7.08, *p* = 1.42 × 10^−5^). We further carried out a set of post-hoc paired two-sample tests to demonstrate the directions of the effect detected. Multiple comparisons of the estimated marginal means based on the three templates in the repeated measures model were also applied. The details of the results are presented in the Table 1. Specifically, for ISYB dataset, both CN200 and Chinese2020 templates exhibited better performances of face masking than the MNI152 template while the 3.0 T MRI derived CN200 template was better than the 1.5 T MRI derived Chinese2020 template. As illustrated in Fig. 6, such effects were highly replicable across the two data subsets (ISYB-1 versus ISYB-2).

**Table 1:** Statistics of the post-hoc tests on the face anonymization with the different templates.

Dataset	Template_1	Template_2	Difference	Standard Error	*p*-value
ISYB	CN200	Chinese2020	1.48 × 10^−3^	5.03 × 10^−4^	9.11 × 10^−3^
ISYB	CN200	MNI152	3.41 × 10^−3^	3.59 × 10^−4^	9.56 × 10^−10^
ISYB	Chinese2020	MNI152	1.93 × 10^−3^	4.67 × 10^−4^	1.12 × 10^−4^

### Guidance and implication

With the continuous development of neuroimaging, the distinction in the brain structure across different races can become more refined. The comparison of templates of different races provides a basis for more in-depth research on the distinctions of separate races. However, many challenges have also emerged, especially when the private information is easily leaked through images. At the same time, due to the continuous development and maturity of deep learning technology, combined with big data, the entire face can be reconstructed and refabricated using a small portion of facial information. This forces us to pay special attention to protection of subjects’ private information, especially facial information. Masking the face of the data by using the face masking toolkit effectively blur the subjects’ faces, thereby protecting privacy. Meanwhile, it protects the integrity of the brain structure, which makes the structure MRI easier to interface with mainstream brain image analysis software. Follow-up analysis is more convenient without degenerating image quality. Since skull shape differs by racial and ethnic groups in length and width^68^, systematic biases may be introduced when images of different races are registered to the same template in the face masking toolkit. This kind of registration difference based on data and templates which are not in the same race has been emphasized in preceding articles, which is inevitable due to genetic factors^41^. This study assumed that using different human brain templates to anonymize facial data would yield different results^69^. We selected 215 data from Chinese database and three brain templates from two races, and used ethnicity-matched and ethnicity-mismatched templates to blur the facial information of the data, explored the impact of different ethnic templates on data anonymization.

After face masking was performed on Chinese data respectively using brain templates from two races, matching the data according to the ethnic template, quantifying the results according to the index of normalized mutual information, it can be concluded that: (1) There existed a significant difference in the anonymization results when the results based on the two Chinese templates were compared with that based on the Western template. The different ethnicity of the templates lead to the differences in the results when using different ethnic templates to blur face information in structural brain images by the face masking toolbox. Therefore, we believe that such variability is attributed to the race factor. (2) Choosing a template that matches the race of the data will achieve better results in comparison to with a mismatched template when using different templates to anonymize data. In other words, the template selected during anonymization should correspond to the race of the data so that better private information anonymity results can be achieved. In the process of masking the face, the surface mask algorithm proposed by Budin and colleagues was used and performed^70^. The face area was identified and positioned, and then smoothed by using a blur algorithm to obtain a contaminated face image. However, in the face mask algorithm, the data need to be registered on the specific template, and then the brain tissue position and skull boundary information could be found through the binary image, thereby blurring the part between the brain and skull. The registration effect varies from race to race, resulting in different positions of facial features and causing disparate phenomena based on different templates when using templates of different races as a reference to registration.

The effect of facial anonymization varies by brain templates to a large extent, the results demonstrate that there exist a difference in the effect of facial masking based on two Chinese templates, the difference may attributed to the fact that the two Chinese templates are constructed using data obtained with different field strengths (1.5 T and 3.0 T). What we want to emphasis is that the anonymization effect is determined by the ethnicity of the templates. The brain structure of individuals from different ethnic group is quite different. Although different templates can be used to anonymize structure MRI data, the use of ethnicity-matched templates will lead to the best anonymization effect. This also demonstrated that the accuracy of brain image data processing could be enhanced by using the ethnicity-appropriate templates. Improved facial anonymization, conserving less information, has been demonstrated when the Chinese head template is applied to obscure the faces of data acquired in a Chinese population. The customized pipeline using a native Chinese head template is thus recommended for the anonymization of sharing Chinese neuroimaging data. International data-sharing and the relevant large-scale team work on brain imaging such as building brain charts for the human lifespan (see a recent review^21^ and progress^71^) must consider this effect carefully. Its impact on the generalizability of brain-mind association studies^10,72^ is also an interesting topic in the future.

## Supplementary information

Supplementary Figures

## Data Availability

All the scripts and brain templates involved for processing is publicly available as part of the recent CCS updates^57^ and can be visited at GitHub (https://github.com/zuoxinian/CCS/tree/master/projects/isybdemo).
