# Supplementary material for: A Chinese multi-modal neuroimaging data release for increasing diversity of human brain mapping
Source: Sci Data. 2022 Jun 9;9:286. doi: 10.1038/s41597-022-01413-3 (PMC9184635; doi:10.1038/s41597-022-01413-3)
Supplement: Supplementary file 1 — Supplementary Figures [file 41597_2022_1413_MOESM1_ESM.pdf]

A Chinese multi-modal neuroimaging data release for increasing diversity of human brain mapping

Peng Gao[1,†], Hao-Ming Dong[2,3,†], Si-Man Liu[4], Xue-Ru Fan[2], Chao Jiang[5], Yin-Shan Wang[2], Daniel Margulies[6], Hai-Fang Li[1,*], and Xi-Nian Zuo[2,3,4,7,8,*]

[1]College of Information and Computer, Taiyuan University of Technology, Taiyuan, 030024, China
[2]State Key Laboratory of Cognitive Neuroscience and Learning, Beijing Normal University, Beijing, 100875, China
[3]National Basic Science Data Center, Beijing, 100109, China
[4]Institute of Psychology, Chinese Academy of Sciences, Beijing, 100101, China
[5]School of Psychology, Capital Normal University, Beijing, 100048, China
[6]Centre National de la Recherche Scientifique, Frontlab, Brain and Spinal Cord Institute, Paris, UMR 7225, France
[7]Developmental Population Neuroscience Research Center, IDG/McGovern Institute for Brain Research, Beijing Normal University, Beijing, 100875, China
[8]Key Laboratory of Brain and Education, School of Education Science, Nanning Normal University, Nanning, 530001, China
[*]**corresponding author(s)**: Hai-Fang Li (lihaifang@tyut.edu.cn) and Xi-Nian Zuo (xinian.zuo@bnu.edu.cn)
[†]**these authors contributed equally to this work**

ABSTRACT

This document contains the supplementary information including the two quality control reports generated by **MRIQC** for the ISYB dataset.

Supplementary information

MRIQC: ISYB group T1w report

Summary

- Date and time: 2021-03-22, 06:48.
- MRIQC version: 0.16.1.

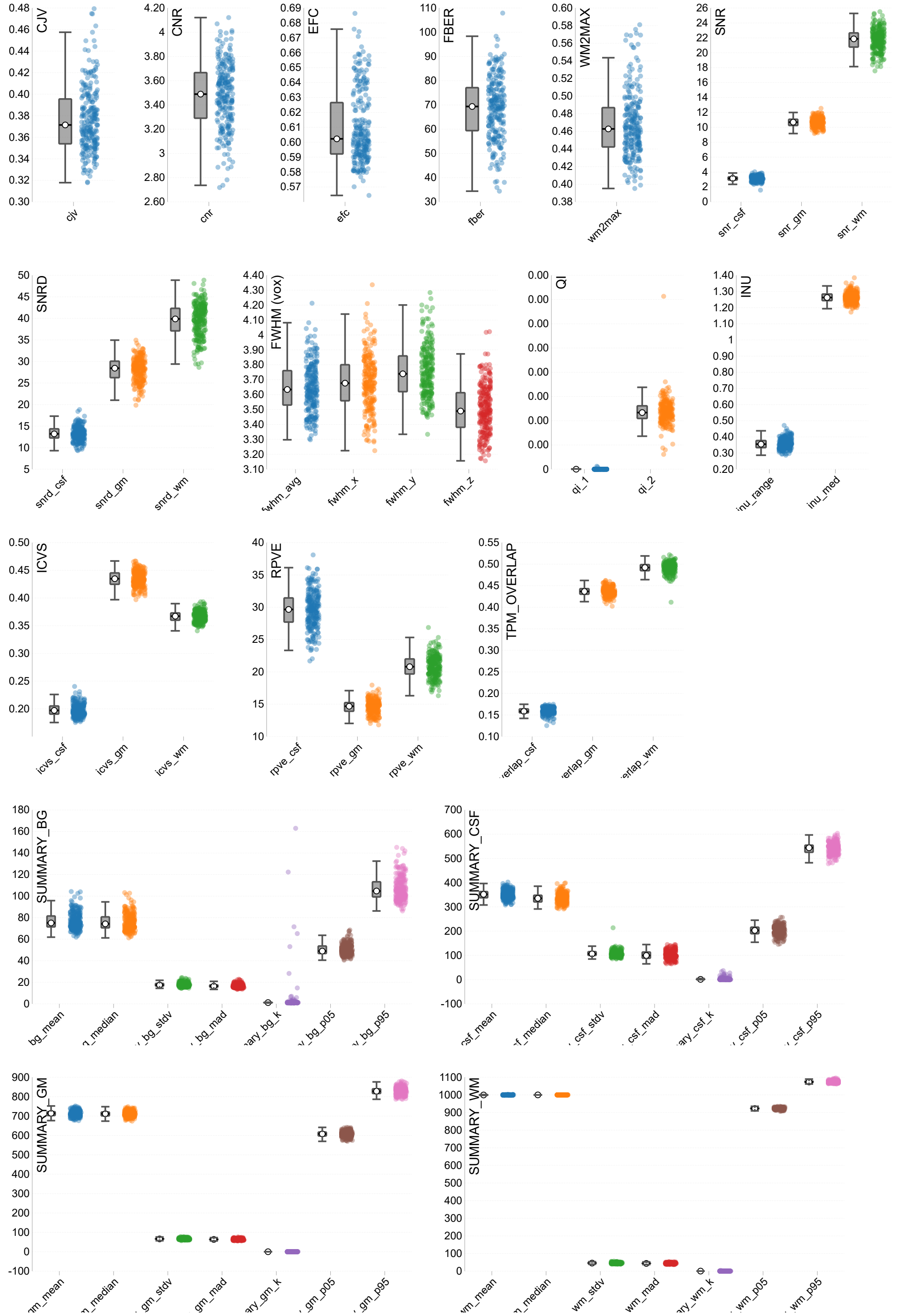

SFigure 1. Group level structural (T1w) MRI quality control report generated by **MRIQC** for each subjects in ISYB dataset.

MRIQC: ISYB group bold report

Summary

- Date and time: 2021-03-22, 06:48.
- MRIQC version: 0.16.1.

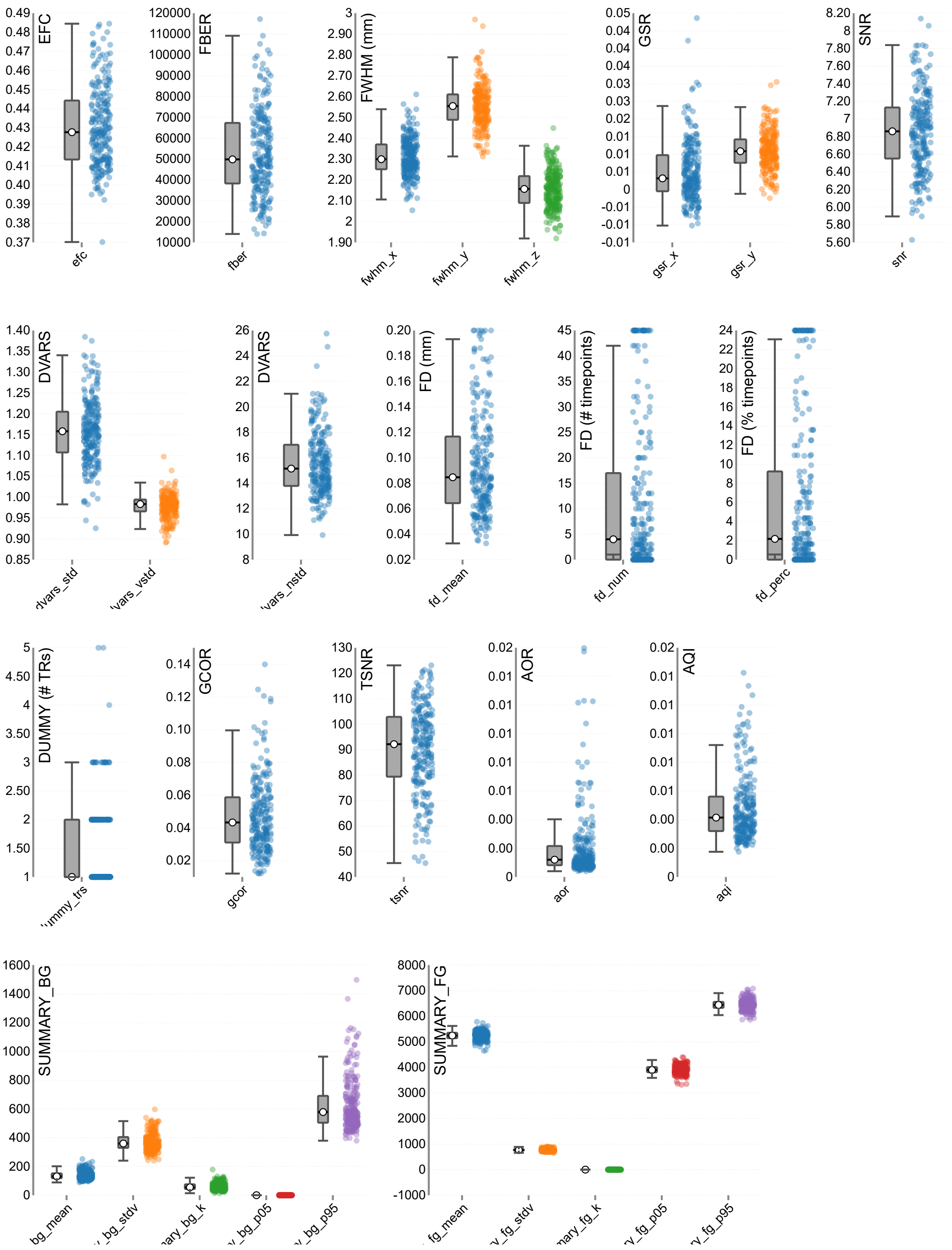

SFigure 2. Group level functional (BOLD) MRI quality control report generated by **MRIQC** for each subjects in ISYB dataset.
